# Decorin Protects Retinal Pigment Epithelium Cells from Oxidative Stress and Apoptosis via AMPK-mTOR-Regulated Autophagy

**DOI:** 10.1155/2022/3955748

**Published:** 2022-03-29

**Authors:** Xinyi Xie, Duo Li, Yuqing Cui, Tianhua Xie, Jiping Cai, Yong Yao

**Affiliations:** Department of Ophthalmology, Wuxi People's Hospital Affiliated to Nanjing Medical University, Wuxi, Jiangsu, China

## Abstract

Age-related macular degeneration (AMD) is the leading cause of irreversible visual loss among the elderly worldwide with unidentified pathogenesis and limited therapeutic options. Oxidative stress-induced damage to the retinal pigment epithelium (RPE) is central in the development and progression of AMD. Decorin (DCN), a small leucine-rich proteoglycan, possesses powerful antifibrotic, anti-inflammatory, and antiangiogenic properties. DCN has also been reported to serve a cytoprotective role in various cell types, but its protective effects against H_2_O_2_-induced oxidative stress and apoptosis in ARPE-19 cells remain unclear. In this study, we showed that DCN significantly attenuated the increase in cell viability loss, apoptosis rate, and reactive oxygen species (ROS) levels in ARPE-19 cells induced by H_2_O_2_. Furthermore, DCN activated the AMPK/mTOR pathway to promote autophagy while genetic inhibition of autophagy-related gene 5 (ATG5) hindered autophagic process and diminished the protective role of DCN against oxidative stress in ARPE-19 cells. Collectively, these results suggest that DCN could protect RPE cells from H_2_O_2_-induced oxidative stress and apoptosis via autophagy promotion, thus providing the therapeutic potential for AMD prevention and treatment.

## 1. Introduction

Age-related macular degeneration (AMD) is a chronic, progressive neurodegenerative disease that affects older individuals and features loss of central vision. AMD accounts for 8.7% of all blindness worldwide and is the leading cause of irreversible vision disability among older adults in developed countries. By 2040, it is expected that 288 million individuals will be diagnosed with AMD [1]. Broadly, AMD can present in three clinical forms: early nonexudative (dry), late nonexudative (atrophic), and exudative or neovascular (wet). Early AMD is characterized by the presence of drusen in the aging macula without visual loss [2]. The vast majority of AMD patients suffer from dry or atrophic AMD, characterized by the accumulation of drusen, the degeneration of RPE cells and photoreceptors, and geographic atrophy [3]. Choroidal neovascularization is accompanied by an advanced form of wet AMD, leading to exudation and bleeding within the macula. Therapeutic intervention for patients with wet AMD involves repeated administration of intravitreal antivascular endothelial growth factor (VEGF) [4]. However, currently, there is no intervention to slow the progression of the early stages of AMD, and no definitive treatment is available for nonexudative AMD due partly to our incomplete understanding of the underlying pathogenesis of AMD.

AMD is a highly complex neurodegenerative disease that is affected by a combination of aging, genetic, and environmental stressors. The exact pathogenesis of AMD remains unknown [5, 6]. Aging is the primary risk factor associated with AMD, as this irreversible process is accompanied by accumulated oxidative stress, chronic inflammation, and mitochondrial dysfunction [7]. Among these risk factors, numerous studies have shown that the accumulation of oxidative stress may lead to morphological and functional abnormalities in retinal pigment epithelial (RPE) cells, which is the core pathophysiological change in AMD [8, 9]. In physiological processes, normal active retinal metabolism involves high oxygen levels and promotes the generation of reactive oxygen species (ROS), leading to a highly oxidative environment in which the RPE is exposed [10, 11] Dysfunction of the antioxidant system and other pathological factors during aging contributes to ROS overaccumulation, resulting in oxidative cell damage in RPE cells [12].

Autophagy is particularly important for maintaining the homeostasis of the RPE because these cells are exposed to sustained oxidative stress [13]. Several studies have reported that autophagy plays a vital role in the antioxidative process and promotes the clearance of damaged organelles or misfolded proteins in RPE cells during oxidative stress [14–16]. Autophagy dysfunction has also been associated with pathogenesis of AMD [17]. Numerous studies have demonstrated that autophagy activators such as melatonin and rapamycin showed great potential in AMD treatment [18, 19]. Among these activators, decorin (DCN) has been shown to promote autophagy in various cells including endothelial, nucleus pulposus, and breast carcinoma cells [20–23]. DCN, identified as a class I small leucine-rich proteoglycan (SLRP), localizes to the cornea, skin, and other connective tissue and serves as a regulator of various cellular function by fettering to extracellular matrix molecules or through cell receptors [24]. The biological function of DCN mainly involves cell growth, differentiation, and proliferation through its interaction with various molecules, such as transforming growth factor-beta and insulin-like growth factor receptor 1 [25, 26]. A previous study demonstrated that DCN showed an inhibitory effect on oxidative stress and apoptosis in posttraumatic brain injury in rat cerebrum as depicted by increased activity of superoxide dismutase (SOD) and glutathione peroxidase (GSH) [27, 28]. Moreover, DCN may inhibit glucose-induced oxidative stress and apoptosis of human lens epithelial cells and protect the barrier function of RPE cells under high-glucose conditions [23]. However, the potential protective effects of DCN on AMD have not yet been studied. In this study, we aimed to investigate whether DCN could protect RPE cells from H_2_O_2_-induced oxidative stress and explore the underlying mechanism.

## 2. Materials and Methods

### 2.1. Cell Culture

The ARPE-19 human cell line was purchased from the Cell Bank of the Chinese Academy of Sciences (Shanghai, China). Before the experiment, the cells were seeded at approximately 0.5 × 10^5^/cm^2^ and maintained in culture for two weeks unless otherwise stated to establish a robust oxidative stress model. The cells were cultured in Dulbecco's modified Eagle's/Ham's F-12 medium (DMEM/F12) containing 10% fetal bovine serum (FBS, Gibco, USA) in humidified air with 5% CO_2_ at 37°C. The culture medium was changed every two days.

### 2.2. Cell Viability Assay

The viability of ARPE-19 cells was evaluated using the CCK-8 reagent (Dojindo, Japan) according to the manufacturer's instructions. Briefly, ARPE-19 cells were seeded in 96-well culture plates with 1 × 10^4^ cells per well and treated with H_2_O_2_ and DCN for the indicated times. Subsequently, the culture medium was replaced with medium containing 10% CCK-8 solution at 37°C for 4 h. The absorbance of each well was determined using microplate spectrophotometer (BioTek, USA) at a wavelength of 450 nm. The OD absorbance was normalized to that of the untreated controls.

### 2.3. Measurement of SOD, Malondialdehyde, and GSH

For SOD determination, ARPE-19 cells were treated with different conditions, and the SOD activity was determined using Superoxide Dismutase Assay Kit (Beyotime, China) according to the manufacturer's instructions. Briefly, after the indicated treatment, the cells were washed twice with cold PBS. Subsequently, the sample solution was added to the working solution and incubated at 37°C for 30 min. As a result, the absorbance was determined at the wavelength of 450 nm as describe above. In addition, the content of malondialdehyde (MDA) and glutathione was determined using a commercial assay kit (Beyotime, China) and measured at wavelength of 532 nm and 414 nm, respectively, after incubation with the working solution according to the manufacturer's instructions.

### 2.4. Western Blot

Western blot analysis was conducted as described previously [29]. Briefly, the cells were lysed with buffers containing inhibitor targeting protease and phosphatase. Equal volumes of protein were then subjected to sodium dodecyl sulfate-polyacrylamide gel electrophoresis and transferred to polyvinylidene difluoride membranes (Millipore, USA). Subsequently, the transferred membrane was blocked with bovine serum albumin for 1 h and incubated with the following primary antibodies against ATG5 (60061-1-Ig, PTG, China), LC3 (12741, CST, USA), p62 (5114, CST, USA), BAX (50599-2-Ig, PTG, China), Bcl-2 (12789-1-AP, PTG, China), cleaved-caspase 3 (5A1E, CST, USA), AMPK(5831, CST, USA), p-AMPK(50081, CST, USA), mTOR(2983, CST, USA), p-mTOR(5536, CST, USA), p70S6K(9202, CST, USA), p-p70S6K(97596, CST, USA), and *β*-actin (4970, CST, USA). The membranes were then washed with Tris-buffered saline with 0.1% Tween 20 (TBST) and incubated with the horseradish peroxidase-conjugated secondary antibodies (7074, CST, USA). The membranes were then washed and visualized using a chemiluminescence system with an ECL reagent (Yeasen, China).

### 2.5. mRFP-GFP-LC3 Transduction and Analysis

Before DCN treatment, ARPE-19 cells were transduced with Ad-mRFP-GFP-LC3 (HanBio, China) at five multiplicities of infection for 3 h according to the manufacturer's instructions. The autophagosomes and autolysosomes in ARPE-19 cells were visualized using a Leica SP5 confocal laser scanning microscope (CLSM) and quantified using ImageJ software.

### 2.6. Transmission Electron Microscopy

ARPE-19 cells were cultured in 6 cm dishes and subjected to different treatments. The cells were washed carefully and collected by scrapping. Then, the cells were collected after centrifugation and fixed in 2.5% glutaraldehyde solution for 24 h at 4°C. Subsequently, the ARPE-19 cell pellets were hydrated with acetone and embedded in Epon. Ultrathin sections were cut and the cellular structure was observed using a transmission electron microscope (Hitachi, Japan).

### 2.7. Cell Transfection with ATG5 siRNA

ARPE-19 cells were seeded in 6-well plates and transfected with ATG5 siRNA or nontargeting control siRNA at a concentration of 50 nM by Lipofectamine PLUS reagent (Invitrogen, USA) according to the manufacturer's instruction. The transfected ARPE-19 cells were then subjected to different treatments.

### 2.8. Immunofluorescence Staining

ARPE-19 cells were cultured in a confocal dish. To evaluate intracellular ROS, the cells were incubated with 2′,7′-dichlorodihydrofluorescein diacetate (DCFH-DA, Sigma, USA) probes for 30 min at 37°C according to the manufacturer's instructions. Then, the incubated cells were washed with three times PBS and visualized by CLSM. For terminal deoxynucleotidyl transferase dUTP nick end labeling (TUNEL) staining, the cells were fixed with 4% paraformaldehyde for 15 min and washed with PBS three times. Finally, the cells were incubated with 0.1% Triton X-100 for 5 min and stained with the In Situ Cell Death Detection Kit (Beyotime, China) according to the manufacturer's instructions. Then, the cells were visualized by CLSM.

### 2.9. Fluorescence-Activated Cell Sorting Analysis (FACS)

For quantitative evaluation of intracellular ROS, ARPE-19 cells were stained with DCFH-DA as described above. ARPE-19 cells were collected and analyzed by flow cytometry (BD FACS Aria III, USA). For apoptosis evaluation, the cells exposed to different treatments were washed with cold PBS and incubated with the Annexin V-FITC Apoptosis Detection Kit (BD, USA). Stained cells were washed and then collected by trypsin. Then, the cell pellets were resuspended by cold PBS and analyzed by flow cytometry.

### 2.10. Statistical Analysis

All data is presented as mean ± standard deviation (SD) from the results obtained from at least three independent experiments. One-way analysis of variance (ANOVA) with Dunnett's multiple comparisons test was used for statistical analysis. Difference with *p* value less than 0.05 was considered statistically significant. GraphPad Prism 8 was used for the statistical analysis.

## 3. Results

### 3.1. DCN Protected ARPE-19 Cells against H_2_O_2_-Induced Oxidative Cell Death

**Figure 1:**
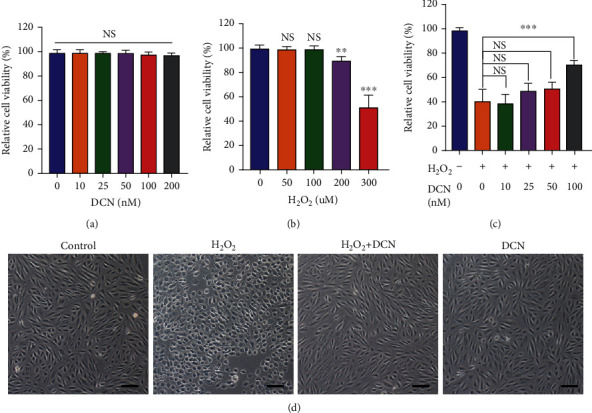
DCN exhibited a protective effect against H_2_O_2_-induced cell damage. (a and b) ARPE-19 cells were treated with DCN or H_2_O_2_ for 24 h at different concentrations and the cell viability was assessed using CCK8. (c) ARPE-19 cells were pretreated with DCN for 24 h at various concentrations and incubated with H_2_O_2_ (300 *μ*M) for another 24 h to determine the cell viability. (d) Morphological changes in ARPE-19 cells following indicated treatment. Data is shown as mean ± SD, ^∗∗^*p* < 0.01, and ^∗∗∗^*p* < 0.001.

In this study, H_2_O_2_ was used to induce oxidative stress, which plays a vital role in the pathology of AMD. As it has been reported that ARPE-19 cells are less vulnerable to oxidative stress with over 1-week longer culture periods, all experiments were conducted after a 2-week cell culture to establish a vigorous oxidative stress model for significant cell damage and death [30]. First, we examined the potential cytotoxicity of DCN in ARPE-19 cells. No significant changes in cell viability were observed after incubation with DCN for 24 h at concentrations of 10–200 nM (Figure 1(a)). Then, to evaluate the protective effect of DCN, ARPE-19 cells were exposed to different concentrations of H_2_O_2_ for 24 h. As shown in Figure 1(b), ARPE-19 cells endured H_2_O_2_ incubation at concentration of 50–100 *μ*M, and the cell viability decreased in a dose-dependent manner at 200 and 300 *μ*M. Furthermore, treatment with 300 *μ*M H_2_O_2_ inhibited cell viability by approximately 50%. Based on these results, ARPE-19 cells were first subjected to DCN at various concentrations for 24 h and then incubated with H_2_O_2_ (300 *μ*M) for another 24 h. Dramatically, DCN significantly attenuated H_2_O_2_-induced cytotoxic effect as demonstrated by 75% increase in the cell viability when ARPE-19 cells were cotreated with DCN at the concentration of 100 nM (Figure 1(c)). Morphological changes in the ARPE-19 cells were also assessed. Incubation with H_2_O_2_ led to apparent cellular changes such as cellular shrinkage. However, coincubation with DCN diminished these morphological changes induced by H_2_O_2_ (Figure 1(d)).

### 3.2. DCN Inhibited H_2_O_2_-Induced Oxidative Stress in ARPE-19 Cells

**Figure 2:**
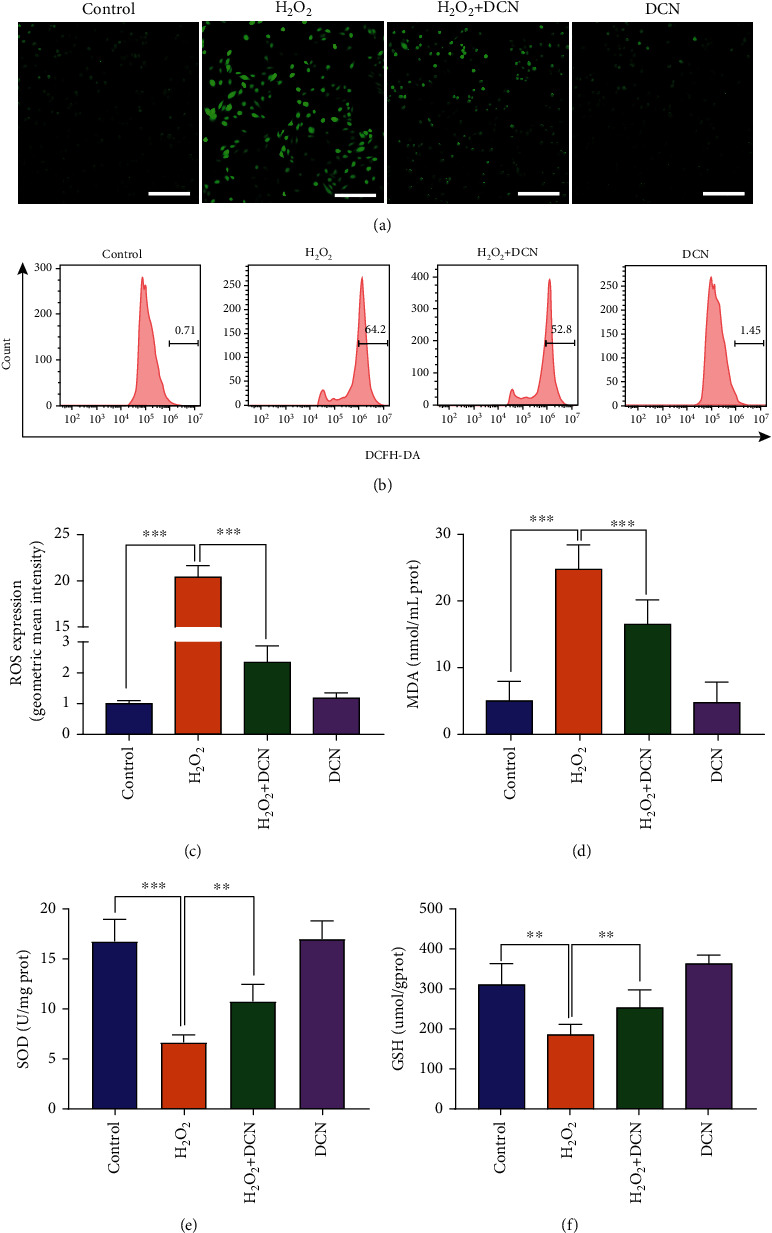
DCN inhibited H_2_O_2_-induced oxidative stress. (a) CLSM images of ARPE-19 cells using ROS detection probes as indicators. (b and c) ROS analysis with flow cytometry and quantitative analysis of ROS fluorescence intensity. (d–f) DCN treatment reversed the H_2_O_2_-induced changes in the level of MDA, SOD, and GSH. Data is shown as mean ± SD, ^∗∗^*p* < 0.01, and ^∗∗∗^*p* < 0.001.

To evaluate the effects of DCN on oxidative stress, ROS generation was assessed. As shown in Figure 2(a), H_2_O_2_ induced a significant increase in ROS expression. Comparatively, cotreatment with DCN remarkably decreased the upregulated level of ROS induced by H_2_O_2_. Similar trends were observed in the quantitative assessment of ROS by flow cytometry (Figures 2(b) and 2(c)). MDA, a lipid peroxidation product, is a kind of biomarkers for oxidative damage [31]. The MDA level in ARPE-19 cells was significantly increased (-4.95-fold) upon H_2_O_2_ treatment, but this increase was notably blocked by DCN treatment (Figure 2(d)). Oxidative stress arises due to redox imbalance between oxidant and antioxidant systems of the cells. SOD and GSH are viewed as important parts in a sophisticated antioxidative defense system to prevent cellular damage by scavenging ROS in the cells [32]. We found that the H_2_O_2_ stimulation decreased the level of both SOD and GSH by 58% and 43%, respectively, compared with the untreated control, while pretreatment with DCN attenuated the reduction induced by H_2_O_2_ (Figures 2(e) and 2(f)).

### 3.3. DCN Ameliorated Apoptotic Cell Death in ARPE-19 Cells under Oxidative Stress

**Figure 3:**
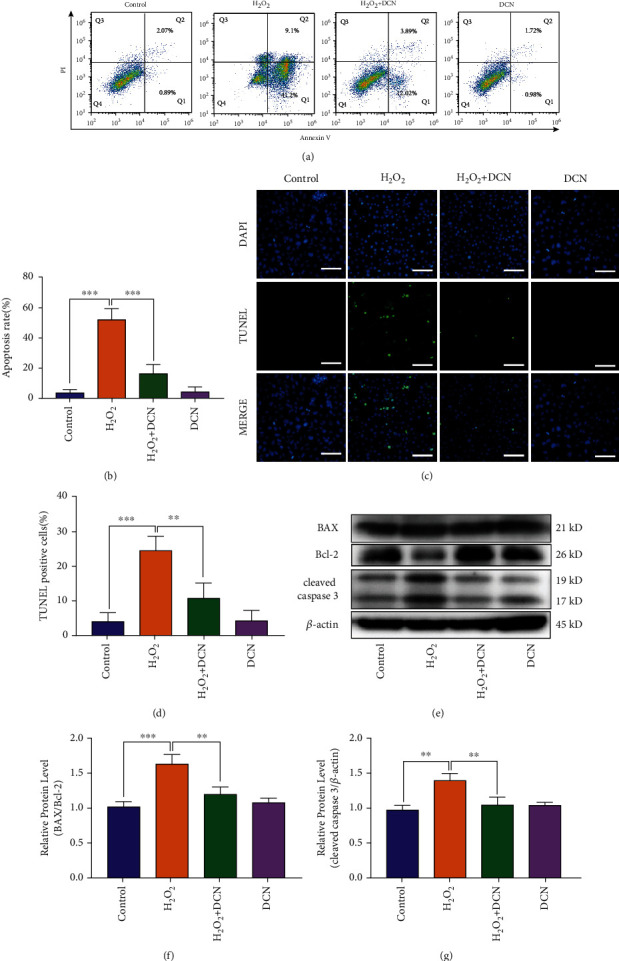
DCN ameliorated the cell apoptosis in oxidative stress. (a) Representative flow cytometry plots using Annexin V-FITC/PI staining for apoptosis in ARPE-19 cells. (b) Quantification of apoptotic cells. (c) TUNEL staining was applied to measure apoptosis levels in ARPE-19 cells. (d) Quantification of TUNEL staining. (e–g) Western blot analysis and quantitative analysis of BAX, BCL2, and Cleaved-Caspase 3 protein level in ARPE-19 cells. Data is shown as mean ± SD, ^∗^*p* < 0.05, ^∗∗^*p* < 0.01, and ^∗∗∗^*p* < 0.001.

To evaluate whether DCN could protect ARPE-19 cells from apoptotic cell death, we determined the apoptosis by flow cytometry, immunofluorescence, and western blot. As shown in Figures 3(a) and 3(b), the population of apoptotic cells was significantly increased by 13.9-fold under oxidative stress conditions. However, treatment with DCN exhibited a significant inhibitory effect on apoptosis of ARPE-19 cells induced by H_2_O_2_. In addition, we performed TUNEL staining and similar results were observed (Figures 3(c) and 3(d)). The Bcl-2 (B-cell lymphoma-2) family proteins play an important role in regulating the mitochondria-dependent extrinsic and intrinsic cell apoptosis. This family of proteins is mainly divided into various antiapoptotic and proapoptotic categories, among which antiapoptotic Bcl-2 and proapoptotic BAX (Bcl-2 Associated X-protein) play a central role in regulating apoptosis [33]. Subsequently, western blot was applied to evaluate the Bax/Bcl-2 ratio accompanied by the downstream protein cleaved caspase-3. As shown in Figures 3(e)–3(g), western blot results indicated that H_2_O_2_ significantly increased the Bax/Bcl-2 ratio and the expression level of cleaved caspase-3 by 60% and 43%, respectively. However, DCN intervention could reverse above effects induced by H_2_O_2_. These results indicated that DCN exerted a protective effect against H_2_O_2_-induced oxidative cell damage via antiapoptosis.

### 3.4. DCN Promoted Autophagy in H_2_O_2_-Treated ARPE-19 Cells

**Figure 4:**
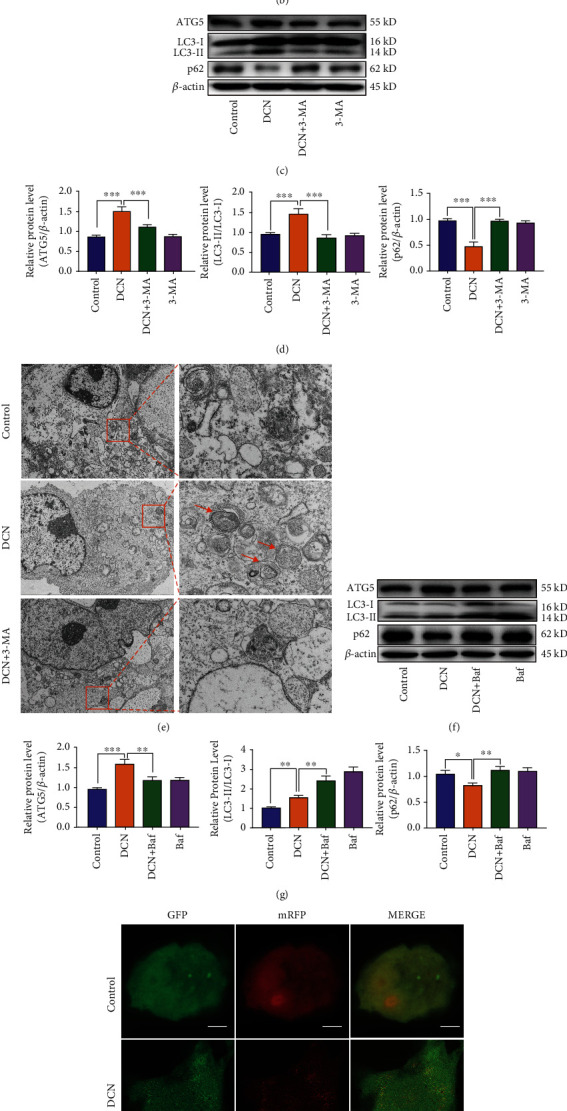
DCN promoted autophagy in ARPE-19 cells. (a and b) The protein level of ATG5, p62, and LC3 in ARPE-19 cells. ARPE-19 cells were treated with DCN in indicated concentrations for 24 h. The densities of ATG5/*β*-actin, p62/*β*-actin, and LC3II/LC3I were analyzed. (c and d) The protein level of ATG5, p62, and LC3 in ARPE-19 cells treated with 100 nM DCN in the presence of 5 mM 3-MA. The densities of ATG5/*β*-actin, p62/*β*-actin, and LC3II/LC3I were analyzed. (e) The ultrastructures in ARPE-19 cells were examined by transmission electron microscopy (TEM). The orange arrow indicates autophagosome and autolysosomes in the cytoplasm. (f and g) The protein level of ATG5, p62, and LC3 in ARPE-19 cells treated with 100 nM DCN in the presence of 0.1 *μ*M bafilomycin A1 (Baf). The densities of ATG5/*β*-actin, p62/*β*-actin, and LC3-II/LC3-I were analyzed. (h) ARPE-19 cells were transfected with mRFP-GFP-LC3 and subjected to 100 nM DCN treatment. Data is shown as mean ± SD, ^∗^*p* < 0.05, ^∗∗^*p* < 0.01, and ^∗∗∗^*p* < 0.001.

Autophagy is intricately linked to apoptosis in various degenerative diseases. Aberrant autophagy has been reported to be associated with AMD. Several lines of evidence have demonstrated that autophagy acts as a mediator in maintaining ROS balance to protect ARPE-19 cells against oxidative stress [13, 15]. To determine whether DCN could activate autophagy in ARPE-19 cells, we examined autophagy proteins by western blot. The results showed that the ATG5 protein expression and LC3-II/LC3-I ratio were increased, accompanied by decreased P62 expression under DCN stimulation in a dose-dependent manner (Figures 4(a) and 4(b)). To further investigate autophagy promotion by DCN, ARPE-19 cells were treated with the autophagy inhibitor 3-methyladenine (3-MA). Treatment with 3-MA partly diminished the autophagy process induced by DCN, as 3-MA decreased ATG5 expression and the ratio of LC3-II/LC3-I by 26% and 41%, respectively, while increased the accumulation of p62 by 104% compared with the treatment with DCN (Figures 4(c) and 4(d)). To confirm the activation of DCN-induced autophagy, transmission electron microscope (TEM) ultrastructural analysis was performed as golden standard for autophagy detection. TEM images showed that an increased number of autophagic vacuoles were observed in DCN-treated ARPE-19 cells, but not in the control cells, and cotreatment with 3-MA inhibited the formation of autophagosome induced by DCN (Figure 4(e) and S1). To further confirm the autophagic changes and validate the autophagic flux induced by DCN, bafilomycin A1 (Baf), an H+-ATPase inhibitor, was used to inhibit the fusion of autophagosomes with lysosomes during autophagy. As shown in Figures 4(f) and 4(g), Baf significantly increased the ratio of LC3-II/LC3-I by 55% and p62 expression by 36% while downregulated ATG5 expression by 26% in DCN-treated ARPE-19 cells compared to treatment with DCN alone. Moreover, we assessed autophagic flux in ARPE-19 cells using adenovirus-mediated mRFP-GFP-LC3. The autophagosomes in transduced ARPE-19 cells were marked by mRFP-GFP-LC3 and exhibited both red fluorescent protein (RFP) and green fluorescent protein (GFP), displayed as yellow dots. In contrast, only red dots were observed in autolysosomes because the green GFP fluorescence was eliminated in the acidic environment of the lysosomes. DCN significantly increased the number of both autophagosomes and autolysosomes compared with control cells, indicating a high level of autophagy flux. However, the number of red autolysosomes declined in the case that Baf was present to blockage of autophagy flux (Figure 4(h) and S2).

### 3.5. DCN Inhibited H_2_O_2_-Induced Oxidative Stress in Autophagy-Dependent Manner

**Figure 5:**
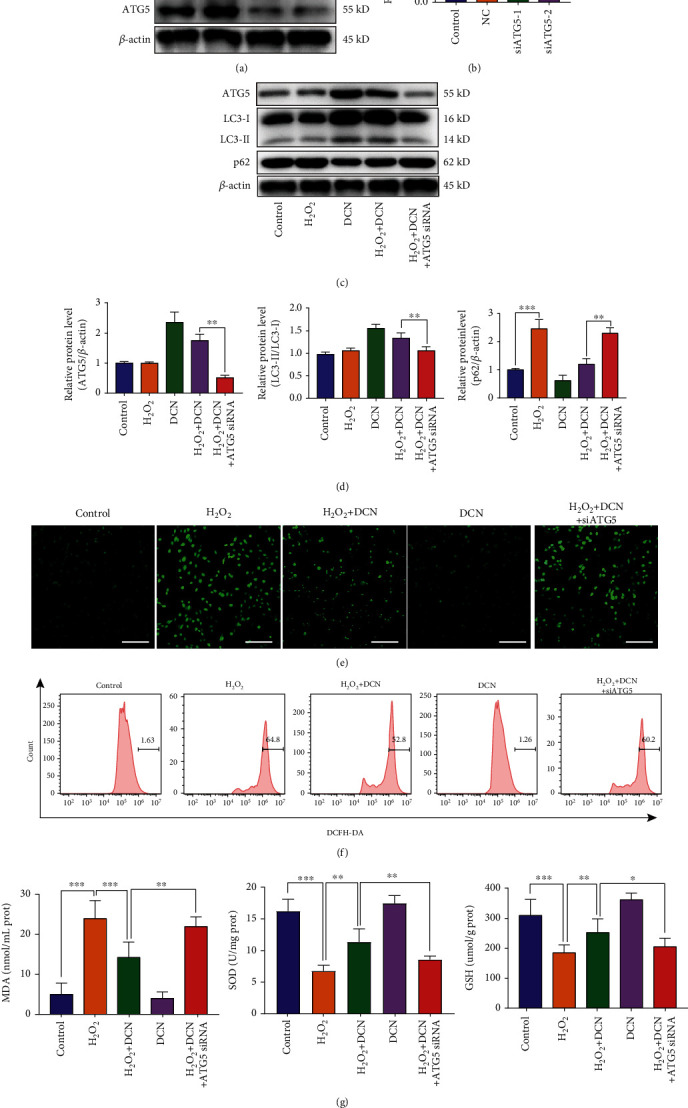
Protective effect of DCN against oxidative stress was dependent on autophagy. ARPE-19 cells were first transfected with ATG5 siRNA and received different treatments. (a and b) The expression level of ATG5 in ARPE-19 cells after ATG5 small interfering RNA (siRNA) transfection. The densities of ATG5/*β*-actin were analyzed. (c and d) The protein levels of ATG5, p62, and LC3 in ARPE-19 cells in the presence of ATG5 siRNA transfection followed by treatment of DCN under the stimulation of H_2_O_2_. (e) CLSM images of ARPE-19 cells using ROS detection probes as indicators. (f) ROS analysis with flow cytometry and quantitative analysis of ROS fluorescence intensity. (g) The expression level of MDA, SOD, and GSH following indicated treatment. Data is shown as mean ± SD, ^∗^*p* < 0.05, ^∗∗^*p* < 0.01, and ^∗∗∗^*p* < 0.001.

**Figure 6:**
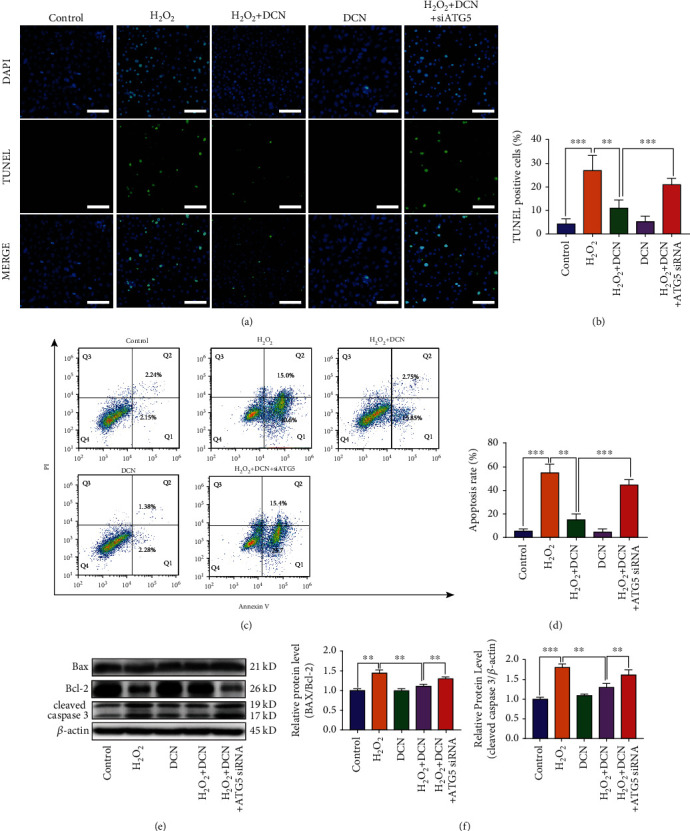
Autophagy inhibition attenuated the protective effect of DCN against cell apoptosis in ARPE-19 cells. (a and b) Microscopic images and quantification of TUNEL-positive ARPE-19 cells. (c) Flow cytometry results with Annexin V-FITC/PI staining. (d) Quantification of apoptotic cells. (e and f) Western blot analysis and quantitative analysis of BAX, BCL2, and Cleaved-Caspase 3 protein level in ARPE-19 cells. Data is shown as mean ± SD, ^∗∗^*p* < 0.01, and ^∗∗∗^*p* < 0.001.

To further investigate whether the protective effect of DCN against oxidative stress was autophagy-dependent in ARPE-19 cells, we next silenced the expression of autophagy-related protein 5 (ATG5), which is indispensable for autophagy. As shown in Figures 5(a) and 5(b), silencing of ATG5 by siRNA resulted in a significant decrease in ATG5 protein expression level. Oxidative stress induced by H_2_O_2_ led to the augmented accumulation of p62 but did not alter the ATG5 protein level and LC3-II/LC3-I ratio. As expected, DCN treatment reduced the expression level of p62 while increased ATG5 expression and LC3-II/LC3-I ratio. However, genetic inhibition of ATG5 diminished the autophagy promotion induced by DCN under oxidative stress (Figures 5(c) and 5(d)). Moreover, ATG5 silencing attenuated the protective effect of DCN against oxidative stress as ROS accumulation was augmented by 109% compared with DCN treatment under H_2_O_2_ stimulation (Figures 5(e) and 5(f) and S3). Similar results were observed for the expression levels of MDA, SOD, and GSH (Figure 5(g)). Also, the antiapoptotic effect of DCN on oxidative stress was prominently impaired by ATG5 knockdown (Figures 6(a)–6(f)).

### 3.6. DCN Induced Autophagy via AMPK-mTOR Pathway

**Figure 7:**
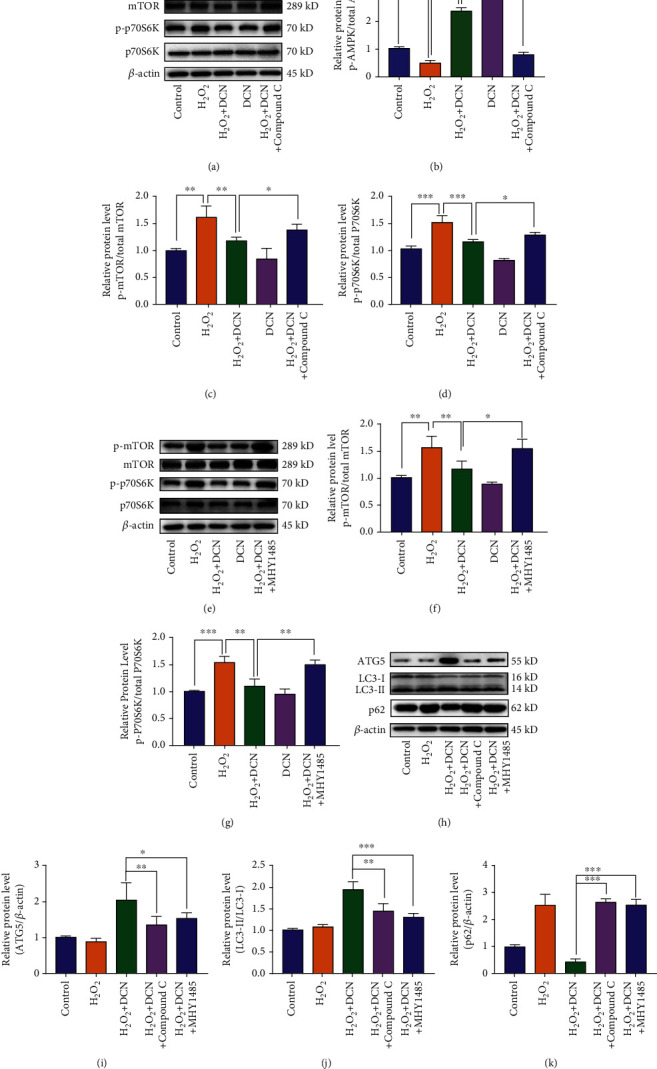
DCN promoted autophagy through AMPK/mTOR signaling pathway. (a–d) The protein levels of p-AMPK, AMPK, p-mTOR, mTOR, p-p70S6K, and p70S6K in ARPE-19 cells. ARPE-19 cells were treated with 5 mM compound C prior to DCN treatment. The densities of p-AMPK/AMPK, p-mTOR/mTOR, and P-p70S6K/p70S6K were analyzed. (e–g) The protein levels of p-AMPK, AMPK, p-mTOR, mTOR, p-p70S6K, and p70S6K in ARPE-19 cells. ARPE-19 cells were treated with 5 *μ*M MHY1485 prior to DCN treatment. The densities of p-mTOR, mTOR, p-p70S6K, and p70S6K were analyzed. (h–k) The protein levels of ATG5, p62, and LC3 in ARPE-19 cells. ARPE-19 cells were treated with 5 mM compound C or 5 *μ*M MHY1485 prior to DCN treatment under oxidative stress. Data is shown as mean ± SD, ^∗^*p* < 0.05, and ^∗∗^*p* < 0.01.

Autophagy is modulated by a variety of signaling pathways. Among these pathways, AMP-activated protein kinase (AMPK)/mammalian target of rapamycin (mTOR) is involved in the regulation of autophagy under oxidative stress [34]. As shown in Figures 7(a)–7(d), H_2_O_2_ stimulation decreased the phosphorylation of AMPK by 52% and increased the phosphorylation of mTOR and its downstream target p70S6K by 18% and 13%, respectively. Treatment with DCN under oxidative stress increased the expression level of p-AMPK by 375% while decreased the phosphorylation level of both mTOR and p70S6K by 27% and 24%, respectively (Figures 7(a)–7(d)). To further elucidate the role of AMPK-mTOR in DCN-induced autophagy, ARPE-19 cells were treated with the AMPK inhibitor compound C or mTOR activator MHY1485 prior to DCN treatment. Compound C inhibited the AMPK-mTOR signaling pathway and antagonized the autophagy promotion by DCN under oxidative stress. Likewise, the mTOR activator MHY1485 was applied to activate mTOR signaling, leading to attenuation of DCN-induced autophagy (Figures 7(e)–7(k)). These results above indicate that autophagy induced by DCN in ARPE-19 cells is dependent on the AMPK-mTOR signaling pathway.

## 4. Discussion

The irreversible visual impairment caused by AMD has become a vital clinical problem, affecting the quality of life among people worldwide [1]. There are two forms of advanced AMD: wet and dry AMD. Currently, the administration of intravitreal anti-VEGF is viewed as a standard therapy with the desired safety and efficacy for wet AMD. Yet there is still no effective therapeutic intervention for dry AMD [6]. Numerous studies have demonstrated that oxidative stress is a key mediator of RPE death or dysfunction in the pathophysiology of AMD [9, 14, 35]. In this manner, approaches aimed at alleviating oxidative stress during AMD progression show promise for treating dry AMD.

In the present study, a reliable *in vitro* cellular model was established, and the protective effect of DCN against oxidative stress was investigated. DCN is a member of the SLRP family and is widely located in various connective tissues, serving as a vital regulator in the physiological processes [21]. Previous study reported that DCN could protect against oxidative stress in glucose-induced lens epithelial cell apoptosis [23]. In the kidney and neuronal tissues, the administration of DCN intraperitoneally ameliorated the MDA level and increased the SOD level [27, 36]. In this study, the viability of ARPE-19 cells decreased by approximately 50% in H_2_O_2_-induced oxidative stress, while pretreatment with DCN increased cell viability and hindered oxidative damage by H_2_O_2_ stimulation, as reflected by decreased MDA and intracellular ROS production. DCN treatment also increased the activity of SOD and GSH in ARPE-19 cells, which was similar to a previous report on the anti-oxidative role of DCN against posttraumatic brain injury [27]. H_2_O_2_-induced oxidative stress also promotes oligomerization of BAX, releases of the apoptosis-related proteins in the cytoplasm, and induced caspase activation, consequently leading to cell apoptosis [37]. In the present study, we found that pretreatment with DCN significantly decreased the expression level of both BAX and cleaved-caspase 3 while increased Bcl-2 expression under oxidative stress. These findings suggest that DCN plays a prominent role in hindering H_2_O_2_-induced oxidative stress and apoptosis in ARPE-19 cells, demonstrating that DCN show potential as a therapeutic approach for dry AMD in the future.

Autophagy is a key process in cellular metabolism and plays a significant role in adaptation to oxidative stress [15]. Downregulated autophagy in RPE cells is associated with the increased susceptibility to oxidative stress in AMD [38]. Previous studies found that DCN could activate autophagy in different kinds of cells including glioma, human hepatoma HepG2, endothelial, and nucleus pulposus cells [22, 23, 39, 40]. To investigate the underlying protective mechanism of DCN against oxidative stress, autophagic markers were evaluated. In the present study, we found that DCN treatment increased autophagy activity in a dose-dependent manner and enhanced the autophagy protein ATG5. ATG5 is essential for the autophagosome precursor and participates in the formation of autophagosome, and loss of ATG5 inhibits autophagy [41]. To further determine whether autophagy participated in the protective effect of DCN against oxidative stress, ATG5 was silenced in ARPE-19 cells to inhibit autophagy. We observed that DCN significantly promoted autophagic flux and protected ARPE-19 cells from oxidative damage, but this effect was mostly diminished after the silencing of ATG5, indicating that DCN protected ARPE-19 cells in an autophagy-dependent manner.

Intriguingly, we noticed a decreased level of autophagy as shown by elevated expression of p62 in H_2_O_2_-treated ARPE-19 cells, which varied from some researchers who hold the opinion that oxidative stress could induce autophagy [18]. In fact, it has also been reported that oxidative stress would either increase or decrease the autophagy activity. In response to transient oxidative stress, autophagy may serve as a self-protective role with increased activity to help rebuild the balance between ROS production and removal. However, oxidative stress in various age-related degenerative diseases, including AMD, tends to be persistent [18, 42]. In this case, autophagy activity was dysregulated, and defective autophagy would finally increase oxidative stress owing to the inability to remove harmful damaged organelles and contribute to AMD progression, which is in line with a previous study on the role of autophagy in osteoarthritis [43].

Furthermore, we investigated the potential molecular mechanisms by which DCN induced autophagy. AMPK-mTOR plays an essential role in regulating autophagy. Autophagy is promoted by AMPK, which is a key energy sensor and an important mediator in maintaining cellular energy homeostasis. Conversely, autophagy is inhibited by mTOR, which plays a key role at the interface of the pathways that coordinately regulate the balance between cell growth and autophagy in response to nutritional status, growth factors, and stress signals [34, 44]. In the process of cellular senescence and aging, such as in RPE cells of AMD patients, decreased phosphorylation of AMPK and overactivated mTOR activity can be observed [45]. To investigate whether AMPK-mTOR signaling was involved in the activation of autophagy following DCN treatment, ARPE-19 cells were first treated with the AMPK inhibitor compound C or mTOR activator MHY1485 prior to DCN treatment. We found that the induction of autophagy by DCN was completely reversed after coincubation with 3-MA or MHY1485. These results indicate that DCN activates AMPK to inhibit mTOR and promotes autophagy in AMPK-dependent manner in ARPE-19 cells, in line with previous studies that observed DCN evoked autophagy by regulating AMPK-mTOR signaling in endothelial, nucleus pulposus, and glioma cells [22, 39, 46].

Emerging evidence indicates that autophagy is a double-edged sword in various physiological and pathophysiological processes [47]. The regulation of autophagy regulation remains complex and has a critical point [48]. In most cases, when the autophagy is mildly activated and the level of autophagy is less than the critical point, autophagy serves as a cytoprotective mechanism by clearing damaged cell components. However, when the autophagy level is above the critical point, overactivation of autophagy may lead to cell death and apoptosis by triggering autophagic cell death pathway and lose cytoprotective function, which is opposite of our goal [47, 48]. To investigate whether prolonged exposure of DCN would exert any cytotoxic effect on ARPE-19 cells, we incubated ARPE-19 cells with DCN for 4 days and found that the treatment of DCN did not exhibit any inhibition on cell proliferation compared with untreated control (Figure S4), which imply that DCN would not induce cytotoxicity after prolonged exposure. The possible mechanism may be related to a negative feedback mechanism termed as autophagic lysosome formation (ALR).

ALR reserves over activated autophagy and restores lysosome homeostasis, coupling the induction and cessation of autophagy [49, 50]. Previous study has demonstrated that ALR requires the activation of mTOR. The reactivation of mTOR initiates ALR to replenish the lysosomal pool for preservation of lysosome homeostasis [50, 51]. In our study, although the activation of mTOR decreased following DCN stimulation for short period, the phosphorylation of mTOR gradually restored to baseline during prolonged DCN treatment (Figure S5), implying that DCN served as an important role in maintaining lysosome homeostasis during long-term incubation by reactivating mTOR. However, more comprehensive studies are needed to clarify the exact role of DCN on the regulation of lysosome biology during autophagy.

## 5. Conclusions

Taken together, we demonstrated that DCN had protective effects against oxidative stress and apoptosis by promoting autophagy in ARPE-19 cells mediated by AMPK-mTOR signaling. Our study not only provided a new insight into the pharmacological mechanism of DCN against oxidative stress but also supported therapeutic potential of DCN in the prevention and treatment of AMD.

## Data Availability

The raw data supporting the conclusions of this article will be made available by the authors upon reasonable request, without undue reservation.

## Supplementary Materials

Supplementary Materials Figure S1: quantification of autophagosomes in 20 cells for each condition. Data is shown as mean ± SD and ^∗∗∗^*p* < 0.001. Figure S2: ARPE-19 cells were transfected with mRFP-GFP-LC3 and treated with DCN. The number of yellow autophagosomes(R+G-) and red autolysosomes(R+G+) was quantified. Data is shown as mean ± SD and ^∗∗^*p* < 0.01. Figure S3: quantitative analysis of ROS fluorescence intensity. Data is shown as mean ± SD and ^∗∗∗^*p* < 0.001. Figure S4: relative cell viability of ARPE-19 cells after incubation with DCN for indicated times. The cell viability was determined by CCK8. Figure S5: the protein levels of p-mTOR and *β*-actin in ARPE-19 cells. ARPE-19 cells were treated with DCN for indicated time. The densities of p-mTOR/*β*-actin were analyzed. Data is shown as mean ± SD, ^∗^*p* < 0.05, and ^∗∗∗^*p* < 0.001.
