# Supplementary material for: Decorin Protects Retinal Pigment Epithelium Cells from Oxidative Stress and Apoptosis via AMPK-mTOR-Regulated Autophagy
Source: Oxid Med Cell Longev. 2022 Mar 29;2022:3955748. doi: 10.1155/2022/3955748 (PMC8983248; doi:10.1155/2022/3955748)
Supplement: Supplementary Materials — Figure S1: quantification of autophagosomes in 20 cells for each condition. Data is shown as mean ± SD and ∗∗∗p < 0.001. Figure S2: ARPE-19 cells were transfected with mRFP-GFP-LC3 and treated with DCN. The number of yellow autophagosomes(R+G-) and red autolysosomes(R+G+) was quantified. Data is shown as mean ± SD and ∗∗p < 0.01. Figure S3: quantitative analysis of ROS fluorescence intensity. Data is shown as mean ± SD and ∗∗∗p < 0.001. Figure S4: relative cell viability of ARPE-19 cells after incubation with DCN for indicated times. The cell viability was determined by CCK8. Figure S5: the protein levels of p-mTOR and β-actin in ARPE-19 cells. ARPE-19 cells were treated with DCN for indicated time. The densities of p-mTOR/β-actin were analyzed. Data is shown as mean ± SD, ∗p < 0.05, and ∗∗∗p < 0.001. [file 3955748.f1.docx]

**Supporting Information**

**
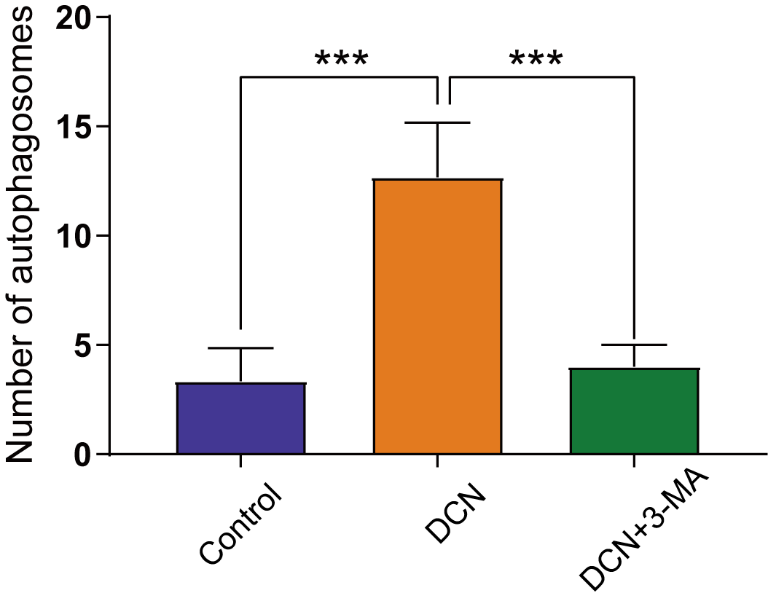
**

**Figure S1.** Quantification of autophagosomes in 20 cells for each condition. Data is shown as mean ± SD, ***p < 0.001.

**
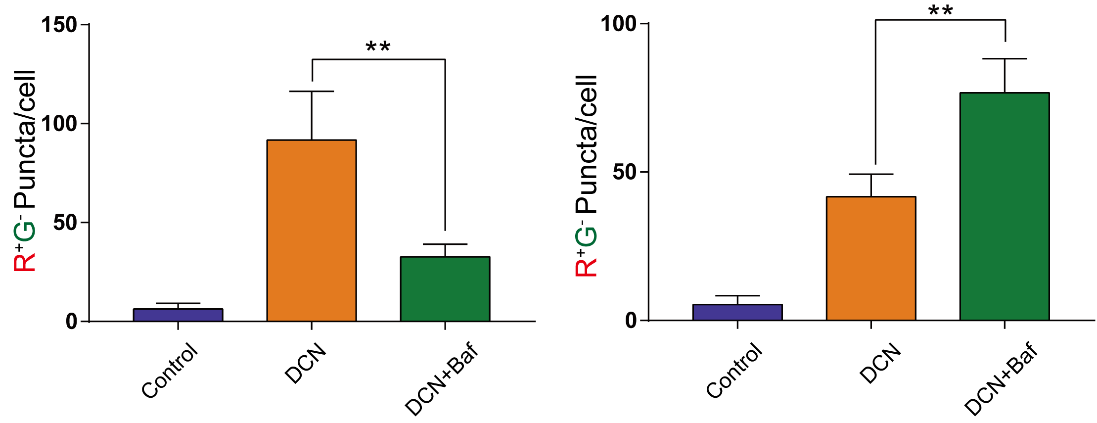
**

**Figure S2.** ARPE-19 cells were transfected with mRFP-GFP-LC3 and treated with DCN. The number of yellow autophagosomes(R+G-) and red autolysosomes(R+G+) were quantified. Data is shown as mean ± SD, **p < 0.01.


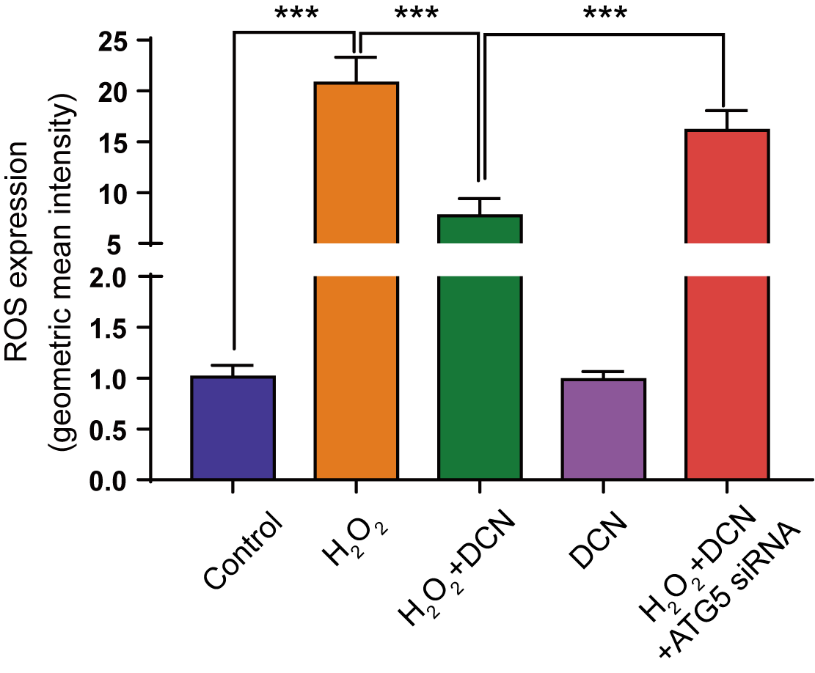


**Figure S3.** Quantitative analysis of ROS fluorescence intensity. Data is shown as mean ± SD, ***p < 0.001.


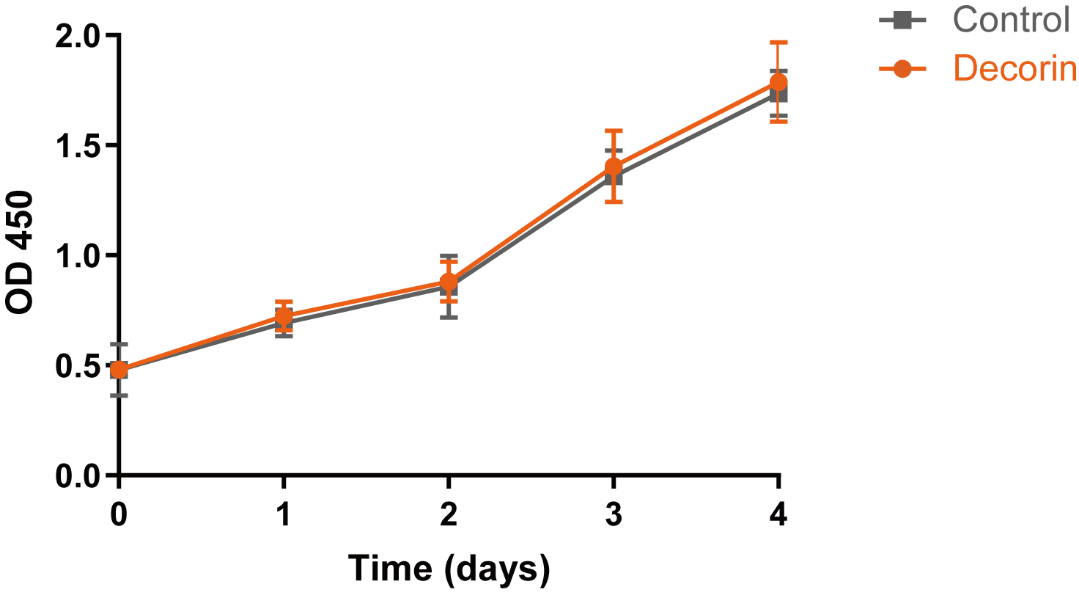


**Figure S4.** Relative cell viability of ARPE-19 cells after incubation with DCN for indicated times. The cell viability was determined by CCK8.

**
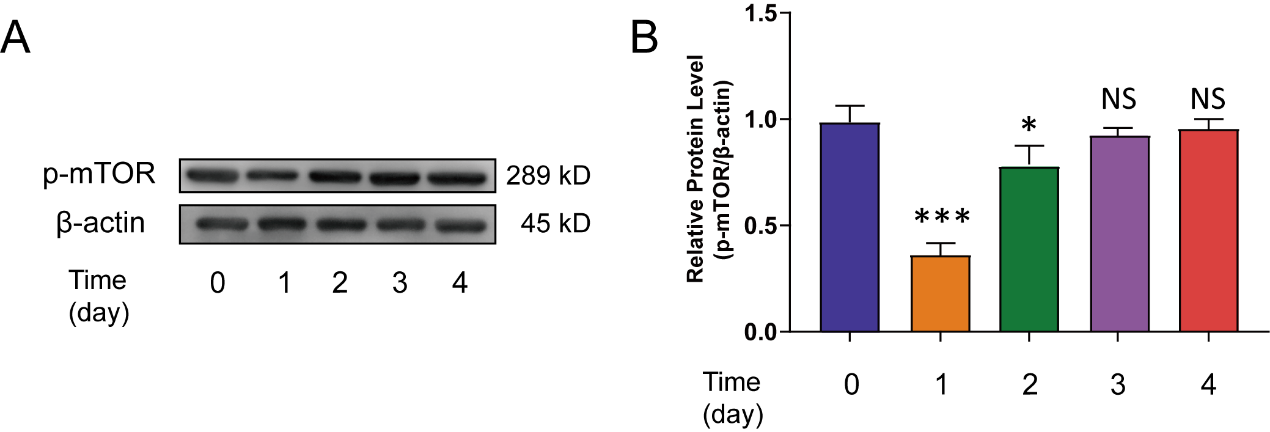
**

**Figure S5.** The protein levels of p-mTOR and β-Actin in ARPE-19 cells. ARPE-19 cells were treated with DCN for indicated time. The densities of p-mTOR/β-actin was analyzed. Data is shown as mean ± SD, *p < 0.05, ***p < 0.001.
